# Does Gender-Fair Language Pay Off? The Social Perception of Professions from a Cross-Linguistic Perspective

**DOI:** 10.3389/fpsyg.2015.02018

**Published:** 2016-01-21

**Authors:** Lisa K. Horvath, Elisa F. Merkel, Anne Maass, Sabine Sczesny

**Affiliations:** ^1^Psychology, University of BernBern, Switzerland; ^2^TUM School of Management, Technical University of MunichMunich, Germany; ^3^Psychology, University of PaduaPadua, Italy

**Keywords:** social perception, gender-fair language, grammatical gender, gender stereotypes, professional groups, status

## Abstract

In many languages, masculine forms (e.g., German *Lehrer*, “teachers, masc.”) have traditionally been used to refer to both women and men, although feminine forms are available, too. Feminine-masculine word pairs (e.g., German *Lehrerinnen und Lehrer*, “teachers, fem. and teachers, masc.”) are recommended as gender-fair alternatives. A large body of empirical research documents that the use of gender-fair forms instead of masculine forms has a substantial impact on mental representations. Masculine forms activate more male representations even when used in a generic sense, whereas word pairs (e.g., German *Lehrerinnen und Lehrer*, “teachers, fem. and teachers, masc.”) lead to a higher cognitive inclusion of women (i.e., visibility of women). Some recent studies, however, have also shown that in a professional context word pairs may be associated with lesser status. The present research is the first to investigate both effects within a single paradigm. A cross-linguistic (Italian and German) study with 391 participants shows that word pairs help to avoid a male bias in the gender-typing of professions and increase women's visibility; at the same time, they decrease the estimated salaries of typically feminine professions (but do not affect perceived social status or competence). This potential payoff has implications for language policies aiming at gender-fairness.

## Introduction

Although women have increasingly entered paid employment in the twentieth century and are making their way up the hierarchical ladders (Eagly and Karau, 2002), there are still considerable gender inequalities in the labor market, as documented by many different indices (e.g., the *Gender Gap Index* of the World Economic Forum, the *Gender Inequality Index* of the UNDP, or the *Social Institutions and Gender Index* of the OECD; for an overview, see European Institute for Gender Equality, 2013). Also, different linguistic forms have been found to contribute to gender-(in)equality: Masculine forms used as generics referring to both women and men lead to a male bias in mental representations. In contrast, feminine-masculine word pairs, which are generally considered gender-fair, increase women's visibility (for an overview, see Stahlberg et al., 2007). This seems to suggest that word pairs promote gender equality. However, recent findings also document detrimental effects of gender-fair language in the professional context, especially on status-related measures (e.g., Formanowicz et al., 2013; Vervecken et al., 2015). These contradictory findings concerning effects of gender-fair language (vs. masculine generics) on gender equality were obtained in different studies under different conditions. The present study is the first to investigate the complex and potentially paradox effects of gender-fair language on the social perception of professional groups within a single paradigm.

### The social perception of professions

Occupational gender-stereotyping follows the proportion of women and men holding the respective professions and translates into the classification of professions as typically feminine and typically masculine (Krefting et al., 1978; Glick, 1991; Glick et al., 1995). The social role theory (Eagly, 1987; Eagly et al., 2000; Koenig and Eagly, 2014) provides a theoretical explanation: Social perceivers' views about social (e.g., occupational) groups and the related stereotypes (e.g., occupational stereotypes) follow from perceivers' experiences and observations of the different distributions of women and men in the respective groups. For instance, when men are observed to occupy the majority of leadership roles, perceivers assume that men possess the traits required for successful leadership, such as decisiveness or dominance (*think-manager—think-male*, Schein, 1973, 2001). On the other hand, individuals occupying certain social roles (e.g., homemaker vs. employee) are described with traits that are stereotypical for these roles (Eagly and Steffen, 1984). Experimental research has confirmed these assumptions. In line with social role theory, occupational stereotyping not only goes back to the observation of typical members of the respective occupational groups, but it actually reflects social reality; additionally, occupational stereotypes can change according to fictitious and varied future job holders despite current stereotypes (Koenig and Eagly, 2014).

A highly relevant variable when dealing with gender in the work place is status. A *gender hierarchy* (Ridgeway and Correll, 2001) continues to be widely prevalent, with men and masculinity being ascribed a higher status than women or femininity. This is mirrored by the following facts: Men are ascribed more competence and worthiness (Ridgeway, 2001), men possess more power, men are more likely to be in leadership positions than women (European Commission, 2011), and men have more access to resources than women (Eagly et al., 2000). Moreover, men receive higher salaries for the same work than women (*Global Gender Gap*, Hausmann et al., 2010). When it comes to the social perception of professions, male-dominated professions are accordingly attributed higher prestige (e.g., Glick et al., 1995). Vice versa, people working in male-dominated professions are assumed to have higher salaries than people working in female-dominated professions, which is indeed the case (Cejka and Eagly, 1999; Alksnis et al., 2008). These gender-status beliefs are consistent with the *Stereotype Content Model* (Fiske et al., 2002): High-status groups (e.g., men) are ascribed higher competence than low-status groups (e.g., women) (Cuddy et al., 2007).

Although gender and status are associated in general, the gender-typing of a profession and professional status/prestige are unrelated dimensions in occupational stereotyping (Gottfredson, 1981, 1996). For instance, the distribution of women and men across professions does not predict estimates of occupational prestige (Glick, 1991). Furthermore, these two dimensions were empirically found to form two independent dimensions in occupational stereotyping (Glick et al., 1995). This is supported by social psychological experiments: Variations in the status of jobs was not reflected in the gender stereotypes ascribed to the job holder (Eagly and Steffen, 1984). This raises the question whether gender-fair language might affect occupational stereotyping concerning both with respect to gender and status.

### Language and gender

Language and cognition are intertwined, with language impacting cognition and vice versa. For instance, color labels and color distinctions that were available in a given language affected native speakers' color perception (Lucy and Shweder, 1979; Winawer et al., 2007) and the order of adjectives and nouns impacts categorization of groups (Percy et al., 2009). With regard to gender, there are differences in how gender is represented in languages (for a detailed overview, see Stahlberg et al., 2007). In *genderless languages* such as Finnish, Turkish, or Chinese gender is mainly expressed through lexical elements of the type “woman,” “man,” “brother,” or “sister.” Otherwise, nouns and pronouns lack gender markings. In *natural gender languages* such as English, Danish, and Swedish as well personal nouns are mostly unmarked for gender, but personal pronouns are gendered. In so-called *grammatical gender languages* like French, Italian or German, additionally to gendered pronouns, all nouns have grammatical gender and many other parts of speech (articles, adjectives, or pronouns that depend on the noun) show grammatical agreement; that means, they signal the gender of the noun. Nouns in these languages are either masculine or feminine, in some languages also neuter (e.g., German). For instance, a table in German is masculine (*der Tisch*, the table, masculine), but feminine in French (*la table*, the table, feminine). Interestingly, the grammatical gender of objects affects the way these objects are perceived: people ascribe more typically masculine (vs. feminine) characteristics to objects that are designated with a grammatically masculine (vs. feminine) noun in their native language (Boroditsky et al., 2003). Thus, gender is in general a highly salient feature in these languages. This also applies to the social perception of professions, where grammatical gender is highly relevant. In languages with grammatical gender, masculine and feminine job titles are available to describe professionals (e.g., German *der Lehrer*, the teacher, masculine/male; *die Lehrerin*, the teacher, feminine/female). Masculine forms, however, are also used as generics (“masculine generics”) to refer to both women and men, to mixed-gender groups or persons whose gender is unknown or irrelevant in a given context (Braun et al., 2005). This traditional use of masculine generics is not considered gender-fair and alternative forms such as feminine-masculine word pairs (German *die Lehrerinnen und Lehrer*, the teachers, fem. and the teachers, masc.) are recommended as replacements (Stahlberg et al., 2007).

Some authors have argued that the existence of grammatical gender in a language is associated with gender (in)equality on a societal level: Gender inequality or gender gaps tend to be bigger in countries with grammatical gender languages (i.e., where masculine forms are used as generics although feminine forms are available) than in countries with natural gender languages or genderless languages. This effect even persists when controlling for religion and political system (Prewitt-Freilino et al., 2012).

Reflecting the latter, there is now ample evidence that the conventional use of masculine forms as generics causes a male bias in mental representations. This effect was replicated and confirmed with different methods in investigations from various disciplines such as social psychology, psycholinguistics or cognitive psychology (see Stahlberg et al., 2007, for an overview). Thus, speakers of German, for instance, associate and retrieve predominantly male exemplars when answering a question with a masculine generic (such as “*Wer ist Ihr Lieblingsmusiker?*” “Who is your favorite musician, masc.?”). In contrast, significantly more female exemplars are mentioned when gender-fair forms are used, such as feminine-masculine word pairs (e.g., *Lieblingsmusikerin/-musiker*, favorite musician, fem./musician, masc.) (Stahlberg et al., 2001; see also Braun et al., 2005).

However, psychological studies on the question whether gender-fair language indeed helps to promote gender equality (masculine generics vs. gender-fair forms) have revealed a complex pattern of effects. Some studies show beneficial effects of gender-fair forms (such as an increased visibility of women), while others describe detrimental effects (such as status loss), as will be discussed below. The present research is the first to investigate this mixed outcome that was observed across different studies within a single paradigm using a multidimensional approach. This is done in order to get a broader picture of occupational stereotyping following gender-fair language and not only a focused view on specific outcomes (Glick et al., 1995). We argue that—for the time being—gender-fair word pairs can simultaneously facilitate and hamper gender equality.

### Linguistic forms and the social perception of professions

Interestingly, language may override widespread stereotypes. For example, according to occupational stereotypes the professions of truck driver or physicist are perceived to be typically masculine; social worker or kindergarten teacher are perceived to be typically feminine professions (Kennison and Trofe, 2003; Irmen, 2007). Even these powerful stereotypes are under the influence of linguistic forms (Irmen and Roßberg, 2004; Braun et al., 2005). In German and French—both grammatical gender languages—but not in English—a natural gender language, where no feminine professional nouns are available—masculine generics caused a male bias in mental associations of professions. Participants assumed more men than women to be in a professional group, even for typically feminine professions. However, the male bias was reduced when respondents were presented with masculine and feminine forms of the respective job titles (Gabriel et al., 2008). Furthermore, a set of studies by Braun and colleagues (2005) showed that various gender-fair forms can help to increase women's visibility in general: word pairs (e.g., German *Musikerinnen und Musiker*, musicians, fem. and musicians, masc.), the capital-I form (*MusikerInnen*, musicians with a capital letter marking the feminine ending as generic and including both women and men), or gender-neutral formulations. But the magnitude of the impact depended on the gender-typicality of professions: When word pairs (vs. masculine forms) were used to refer to a typically masculine profession (geophysicist), more women were assumed to attend a scientific meeting of geophysicists, but this less so for a typically feminine profession (nutritionists). These findings show that linguistic forms have a powerful effect on the social perception of professions and can increase women's visibility.

Linguistic forms also have a tangible impact on behavior in professional contexts. Early research on American English (Bem and Bem, 1973) demonstrated that women and men are more eager to apply for a counter-stereotypical position when the job advertisement refers to both women and men with a gender-neutral form (e.g., *lineworker*) compared to linguistic forms addressing the stereotypical gender. However, use of such gender-specific forms (e.g., *lineman, linewoman*) which were investigated in this study from the 1970s is not permitted any more (UNESCO, 1999). Nevertheless, masculine pronouns (e.g., *he, his, him*) are still used as generics instead of gender-fair forms (e.g., *he/she, her/his, they*). Masculine pronouns—used in reference to an ideal applicant for a vacant position—were found to decrease women's sense of belonging to a professional context, their motivation to pursue the respective position as well as their expected identification with the job compared to gender-neutral forms (*they, the employee*) or word pairs (*he/she, his/her*) (Stout and Dasgupta, 2011). Linguistic forms not only affect potential applicants but also those who make hiring decisions. In a hiring-simulation study on German, decision makers preferred male over female applicants for a high-status leadership position (but not for a middle-management position) when the position was advertised in the masculine (*Geschäftsführer*, CEO, masc.). When word pairs were used (*Geschäftsführerin/Geschäftsführer*, CEO, fem./CEO, masc.), however, women and men were rated as equally suitable for the job (Horvath and Sczesny, 2015).

A number of studies show that children's and adolescents' perceptions of professions and their vocational interests are strongly affected by linguistic forms. For instance, when professions were presented to French adolescents in the masculine, women were perceived to be more successful in typically feminine and men in typically masculine jobs. With word pairs, however, perceptions of success were more balanced: Female and male professionals were perceived as equally likely to succeed in both typically feminine and masculine professions. While linguistic forms did not affect perceived competence, they had an impact on perceived warmth: When professions were presented with masculine forms, holders of typically masculine jobs were perceived as less warm and holders of typically feminine jobs were perceived as warmer compared to the presentation with word pairs. The authors concluded that word pairs shifted perceptions of warmth toward the mid-point and somehow balanced these perceptions, whereas masculine forms tended to evoke gender-stereotypic perceptions of warmth. It should be noted, though, that this was the very first study measuring competence and warmth perceptions of professions (Vervecken et al., 2015). Another study with Belgian and German children showed similar effects for perceptions of success: when professions were presented with word pairs, children estimated female job holders in typically masculine professions as more successful. Furthermore, girls were more interested in these typically masculine professions (Vervecken et al., 2013). However, beneficial as well as detrimental effects of German gender-fair forms have also been observed in children's perception of professions. While word pairs reduced the perceived difficulty of typically masculine professions, and thus increased vocational self-efficacy, they also reduced the estimated salaries (Vervecken and Hannover, 2015).

In a similar vein, use of feminine titles to introduce female professionals in Italian (e.g., *professoressa*, teacher or professor, fem.) instead of masculine titles (e.g., *professore*, teacher or professor, masc.) made these professionals appear less persuasive (Mucchi-Faina, 2005). It has to be noted, though, that this effect may be caused specifically by the feminine suffix *-essa*, as female professionals described with titles ending in *-essa* (corresponding to the suffix *-ess* in English, e.g., *hostess, authoress*) were perceived as having a lower social status than female professionals described with a title ending in -*a* (e.g., *professora*, teacher or professor, fem.), which is a more modern feminine suffix, or with a masculine form (Merkel et al., 2012). Similar disadvantages of linguistic feminization have been described for Polish: Women applying for a gender-neutral job were perceived as less suitable when referring to themselves with a feminine (vs. masculine) professional title (Formanowicz et al., 2013). However, reactions to linguistic forms may change over time, especially as a function of habituation. Thus, when female proponents of social initiatives were introduced with feminine (vs. masculine) forms in Polish, where gender-fair language is relatively new and uncommon, these initiatives were devalued and were not supported. In German, however, where feminine job titles are common, speakers tended to support the initiatives less when female proponents were introduced in the masculine (Formanowicz et al., 2015). Similarly, in Sweden, negative attitudes toward the newly invented gender-neutral personal pronoun *hen*—additionally to the masculine *han* and the feminine *hon*—have been found to diminish over time (Gustafsson Senden et al., 2015). Thus, a feminization of job titles may be detrimental for women when the implementation of gender-fair language starts, but may become integral part of everyday language once speakers have become accustomed to these (initially unfamiliar) forms.

Taken together, past research on the effects of gender-fair language yields a complex pattern: On the one hand, the visibility of women as a group increases when word pairs are used instead of masculine generics (e.g., Braun et al., 2005; Irmen, 2007; Gabriel et al., 2008). On the other hand, a decrease in status-related measures (e.g., social status, salary) is observed when female professionals are introduced with gender-fair (feminine) job titles compared to masculine forms. However, the different studies are based on a wide range of methods and study designs (between- vs. within-participants), which renders a direct comparison difficult. Some studies, for instance, tested one profession only (e.g., Formanowicz et al., 2013), while others included a larger number of professions (e.g., Vervecken et al., 2015). Also, different participant populations (i.e., children, adolescents, students, adults) have been used. Moreover, the effects of gender-fair language have been studied in different languages (e.g., French, Dutch, German, Italian, Polish, English), which have their own structural characteristics. Certain effects may therefore be restricted to the respective language, for instance, negative effects of specific feminine job titles in Italian (e.g., Mucchi-Faina, 2005; Merkel et al., 2012) or Polish (Formanowicz et al., 2013). In some cases, opposing reactions to gender-fair language were found in different languages (e.g., support of social initiatives in German, but rejection in Polish; Formanowicz et al., 2015). Also, some studies focused on the individual level (perception of one person, e.g., Formanowicz et al., 2013), others on the group level (perception of professions, Vervecken et al., 2015). Thus, it is unknown whether gender-fair forms decrease adults' perceptions of professional status on a group level, as is true for children (e.g., Vervecken and Hannover, 2015), and on the individual level (e.g., Mucchi-Faina, 2005; Formanowicz et al., 2013). Most importantly, no study so far has tested whether gender-fair language can *simultaneously* lead to a decrease in perceived status and an increase in visibility. Our aim was, therefore, to test these seemingly contradictory effects within a single paradigm with adult participants.

### Aim and hypotheses

The purpose of the present research was to examine whether gender-fair language pays off by increasing women's visibility or whether it also lowers the perceived status of professions. To answer these questions, we used a repeated measures design in a multidimensional approach and tested the effects of linguistic forms on the perception of professional groups. To increase the generalizability of our findings we investigated two grammatical gender languages, namely Italian and German.

Although gender and status are generally associated (Ridgeway, 2001), gender-typicality and social status might constitute independent and orthogonal dimensions when investigating social perceptions of professions (Glick, 1991; Glick et al., 1995; Gottfredson, 1996). On this basis, we assumed that gender- and status-related measures can indeed simultaneously reveal women's visibility and profession's status loss, even when assessed within a single study.

In our study adults evaluated a list of professions with respect to (a) status-related measures (dimensions that tend to suffer when gender-fair language is used) and (b) women's visibility (a dimension that tend to show greater mental inclusion of women when word pairs are used). The question was whether participants exposed to professions designated with word pairs (e.g., German *Mechanikerinnen und Mechaniker*, mechanics, fem. and mechanics, masc.) would form different perceptions of the respective professional group than those exposed to masculine forms (e.g., German *Mechaniker*, mechanics, masc.). The languages under study were Austrian German and Italian, two grammatical gender languages with structural similarities. Most importantly, professional titles are gender-marked in both languages (e.g., German *Fleischerinnen und Fleischer*; Italian *macellai**e* *e macellai*, butchers, fem. and butchers, masc.), so that we expected comparable findings for the two languages. Moreover, to make results comparable with the most relevant and directly related prior studies (e.g., Braun et al., 2005), we adopted methods and dependent variables from these studies wherever possible. Our hypotheses read as follows:

*Hypothesis 1*: Professional groups are perceived to have a lower social status when designated with word pairs than with masculine forms.

*Hypothesis 2*: Professional groups are perceived to have lower salaries when designated with word pairs than with masculine forms.

*Hypothesis 3:* Professional groups designated with word pairs render women more visible than with masculine forms.

In addition, we examined whether the perceived competence and warmth of the professional groups was also affected. But as there was only one published study with French-speaking children (Vervecken et al., 2015), which had produced rather unexpected findings for warmth, we were reluctant to formulate specific hypotheses. Therefore, perceptions of warmth and competence were investigated in an exploratory way.

## Materials and methods

### Participants

The sample consisted of 391 participants: 195 Austrians (123 women, 72 men; average age 36.03 years, *SD* = 10.53) and 196 Italians (130 women, 66 men; average age 28.55 years, *SD* = 7.42). We recruited participants via snowball sampling and included only individuals over 18 years of age.

### Materials

#### Professions

##### Pretest and selection of target professions

We selected 27 professions (see Appendix A) based on prior research on professional groups (Kennison and Trofe, 2003; Gabriel et al., 2008). Professional titles were selected only when masculine and feminine plural forms were available in both languages, German (e.g., *Dolmetscherinnen und Dolmetscher*, interpreters, fem. and interpreters, masc.) and Italian (e.g., *traduttrici e traduttori*). The web-based pretest on these professions was run with 100 participants (41 Austrians: 26 women, 15 men; 59 Italians: 36 women, 23 men). The dependent variable was gender-typicality of professions (“*Are the following professions more typical of women or men?*”). As in earlier research (Gabriel et al., 2008), answers for each profession were provided on 7-point bipolar scales with the feminine form (e.g., *Dolmetscherinnen/traduttrici*, interpreters, fem.) as one pole (coded as 1) and the masculine form (e.g., *Dolmetscher/traduttori*, interpreters, masc.) as the other pole (coded as 7). Pole labels were counterbalanced across participants: either the feminine or the masculine label appeared on the left end of the scale. Furthermore, we presented the professions in a random order for each participant. Participants filled out the questionnaire in their native language (German or Italian).

Based on these ratings, professions were categorized as typically feminine (< 3.5), gender-neutral (3.5–4.5) or typically masculine (>4.5). In the pretest both Austrian and Italian participants rated seven professions as typically feminine, 13 professions as typically masculine, and three as gender-neutral; judgments of the two national groups were incongruent for four professions (for more details, see Appendix A). For the main study we selected professions on the basis of the following criteria. First, we aimed at including a broad sample of professions, of different gender-typicality but with matching occupational prestige, in order to avoid a prestige-biased sample of professions (as in Glick et al., 1995). Second, we aimed at selecting a comparable number of typically feminine and masculine professions to avoid a statistical bias in the analyses (Tabachnick and Fidell, 2001). Third, we aimed to avoid making the gender-typicality of professions salient. We therefore decided to present not only strongly stereotyped jobs but also additional, more ambiguous professions (seven slightly masculine, three gender-neutral, and four incongruent ones) as fillers in the main study. In order to reduce the questionnaire to a reasonable length, we split the professions into three lists. We selected six of the seven professions judged as typically feminine professions, balanced for occupational prestige as indicated by average salaries (published by Public Employment Service Austria, Arbeitsmarktservice Österreich, 2015). *Six* of the most typically masculine professions were matched with the feminine professions for occupational prestige. The six typically feminine professions selected were *tailors, hairdressers, dancers, interpreters, nutritionists, pharmacists*, and *psychologists*, the last three being rather high in occupational prestige. The six masculine professions selected were: *truck drivers, electricians, mechanics, computer scientists, physicists*, and *engineers* with the last three being rather high in occupational prestige. These 12 final professions were assigned to three lists, whereby each list contained two typically feminine and two typically masculine professions (matched for occupational prestige). Please find a table of the 12 target professions, distributed over the three experimental lists in Appendix B. The filler professions were randomly distributed across the lists and were not included in the main analyses.

#### Linguistic forms

A web-based online questionnaire was used for the main study. Here, all target professions appeared in one of two linguistic versions, namely either in the masculine (e.g., German *Schneider*, Italian *sarti*, tailors, masc.) or in the form of a word pair (e.g., German *Schneiderinnen und Schneider*, Italian *sarte e sarti*, tailors, fem. and tailors, masc.). Each participant was randomly assigned either to the masculine or the word pair condition. If every participant were to rate all professions the questionnaire would have been too long. Therefore, participants were randomly assigned to one of the three lists. In the questionnaire, each profession was followed by a series of items. These items were presented on three separate pages of the online questionnaire. To strengthen the linguistic manipulation, the professions reappeared (in the respective linguistic form) in the heading of each page.

### Dependent variables

We measured the following dependent variables: perceived social status, estimated salary, women's visibility, competence, and warmth, which are described in more detail below. For every dependent variable we aggregated answers for typically feminine and masculine professions separately. Reliabilities for both types of professions are reported below.

#### Perceived social status

The perceived social status of professions was measured with three items developed by Binggeli et al. (2014): (a) “*How much prestige do* [professional group] *have in our society?*” (b) “*How economically successful have* [professional group] *been?*” (c) “*How is the educational level of* [professional group]?” Answers were provided on 7-point bipolar scales (1 = very low; 7 = very high) and item order was randomized. Reliabilities were satisfying for both masculine professions (α = 0.78) and for feminine professions (α = 0.71).

#### Estimated salary

The estimated salary was measured by a single item adopted from Becker et al. (2011): “*Please estimate how much* [professional group] *earn compared to the average Italian / Austrian salary*.” Participants indicated their responses on an 11-point rating scale ranging from −50% (fifty percent below national average) to +50% (fifty percent above national average), in 10% increments. The midpoint represented the national average salary.

#### Women's visibility

Women's visibility was measured with two items which had been used in earlier studies to assess gender typicality: (a) “*How many women and men pursue the profession* [professional group]*?*” (similar to Braun et al., 2005; Gabriel et al., 2008). Answers were provided on an 11-point bipolar scale, ranging from 100% women to 100% men, with 10% increments (90% women, 80% women, 70% women, …); the midpoint was 50% women-50% men; (b) “*For whom is the profession* [professional group] *more typical?*” Answers were provided on a 7-point bipolar scale (ranging from 1 = women to 7 = men, or vice versa). Both items were recoded, so that higher values indicated higher visibility of women. Due to different answering formats we z-standardized the items and merged them. Reliabilities were satisfying for both masculine professions (α = 0.75) and for feminine professions (α = 0.81)

#### Ascriptions of competence and warmth

Ascriptions of competence and warmth were assessed with five items each, adopted from Cuddy et al. (2004) and Cuddy et al. (2009). Participants were asked: “*How would you evaluate* [professional group] *on the following traits? To which degree are they* [competence traits: *able, competent, confident, efficient, skillful*; warmth traits: *warm-hearted, likeable, friendly, altruistic, cordial*]*?*” Answers were provided on 7-point bipolar scales (1 = very little; 7 = very much). The order of the items was randomized. Items for warmth and competence were averaged and reliabilities were satisfying: competence for masculine professions (α = 0.89) and for feminine professions (α = 0.91), warmth for masculine professions (α = 0.91) and for feminine professions (α = 0.93).

An overview of intercorrelations of all dependent variables is provided in Tables 1 and 2, for German and Italian, respectively.

**Table 1:** **Intercorrelations of dependent variables perceived social status, estimated salary, women's visibility, ascriptions of competence and warmth for feminine and masculine professions in German**.

	**1**	**2**	**3**	**4**	**5**
1. Social status	–	0.33^***^	−0.04	0.64^***^	0.42^***^
2. Salary	0.45^***^	–	−0.14	0.15^*^	0.00
3. Women's visibility	−0.17^*^	−0.22^**^	–	0.12	0.17^*^
4. Competence	0.60^***^	0.37^***^	−0.28^***^	–	0.68^***^
5. Warmth	0.40^***^	0.13	−0.06	0.48^***^	–

Intercorrelations for feminine professions are reported above the diagonal, intercorrelations for masculine professions are reported below the diagonal;

* *p < 0.05*,

** *p < 0.01*,

*** *p < 0.001*.

**Table 2:** **Intercorrelations of dependent variables perceived social status, estimated salary, women's visibility, ascriptions of competence, and warmth for feminine and masculine professions in Italian**.

	**1**	**2**	**3**	**4**	**5**
1. Social status	–	0.46^***^	−0.13	0.46^***^	0.31^***^
2. Salary	0.40^***^	–	−0.08	0.22^**^	0.16^*^
3. Women's visibility	−0.03	−0.07	–	0.09	0.09
4. Competence	0.37^***^	0.01	−0.04	–	0.65^***^
5. Warmth	0.26^***^	0.00	0.07	0.48^***^	–

Intercorrelations for feminine professions are reported above the diagonal, intercorrelations for masculine professions are reported below the diagonal;

* *p < 0.05*,

** *p < 0.01*,

*** *p < 0.001*.

### Procedure

Upon entering the web-based questionnaire, participants were informed that the purpose of the study was to investigate social perceptions of various professional groups. In line with APA guidelines (American Psychological Association, 2010), the main instructions at the beginning of the survey included further information, for instance, on expected duration, procedures, and confidentiality. Participants were then presented with nine professional groups. At the end of the questionnaire, they were debriefed and invited to participate in a lottery for gift vouchers, which had been announced at the beginning^1^. The project was approved by the Ethical Committee of the University of Padova in 2010.

## Results

Perceived social status, estimated salary and women's visibility, as well as ascriptions of competence and warmth of typically masculine and feminine professions were analyzed with a 2 (Stereotypicality of Professions: masculine vs. feminine) × 2 (Linguistic Form: masculine forms vs. word pairs) × 2 (Language: German vs. Italian) × 2 (Participant gender: female vs. male) × 3 (List of professions) multivariate analysis of variance (MANOVA) with repeated measures on the first factor. The MANOVA was followed by ANOVAs with pairwise comparisons (with Bonferroni correction) for each dependent variable. Results with *p*-values of 0.05 or less are considered significant. As we were mainly interested in effects of linguistic forms (masculine forms vs. word pairs) and also to enhance readability, we report only those effects that concern our hypotheses: main effects of stereotypicality of professions and effects involving our core factor, linguistic form. All other effects are reported in Appendix C.

The MANOVA revealed a main effect of linguistic form, *F*_(5, 354)_ = 4.57, *p* < 0.001, $\eta_{\text{p}}^{2}=0.06$, indicating that overall masculine forms and word pairs produced different perceptions of professions. No interaction effect involving linguistic form was significant. For all other multivariate effects not involving linguistic form, please see Appendix C.

### Perceived social status

**Figure 1:**
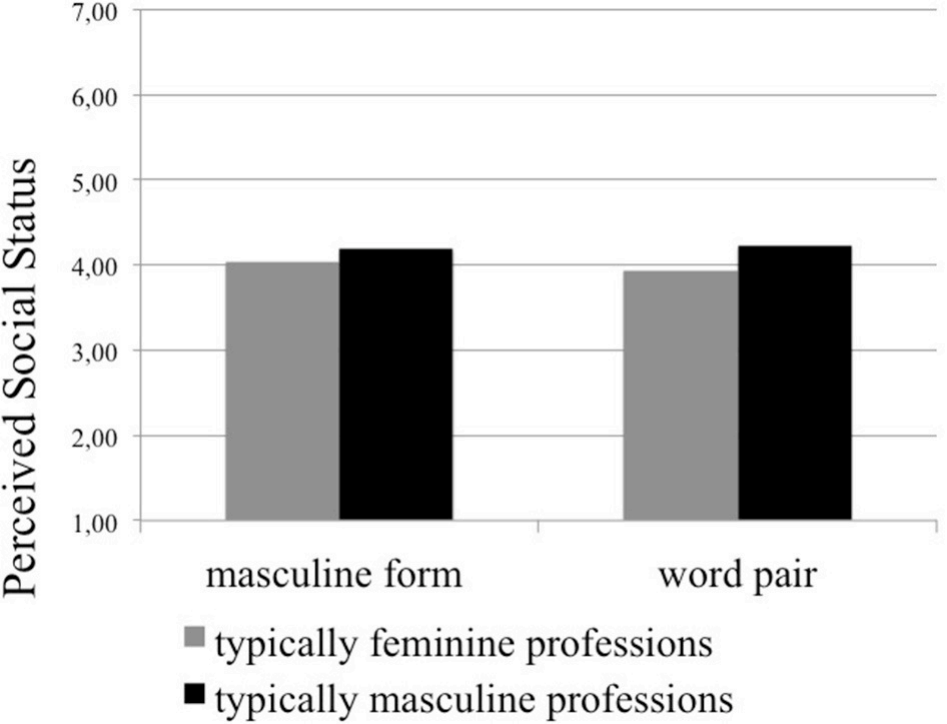
**Perceived social status of typically feminine and masculine professions**. Note that answers were provided on a 7-point scale. Higher numbers indicate higher perceptions of social status.

The ANOVA for perceived social status revealed a significant interaction between stereotypicality of profession and linguistic form, *F*_(1, 363)_ = 4.95, *p* = 0.027, $\eta_{\text{p}}^{2}=0.01$. Pairwise comparisons showed that typically feminine professions were perceived as having lower status than masculine professions, both when presented with masculine forms (*p* = 0.021, η^2^ = 0.02) and with word pairs (*p* ≤ 0.001, η^2^ = 0.07). It is noteworthy that the difference between typically masculine and feminine professions was stronger when word pairs were used. In fact, when word pairs were used, the perceived status of feminine professions declined slightly compared to masculine forms, whereas that of masculine professions increased slightly. These differences are displayed in Figure 1. All means and standard deviations are reported in Table 3. For all other effects not involving linguistic form, please see Appendix C.

**Table 3:** **Means and standard deviations for perceived social status by stereotypicality of professions, linguistic form, list, and participant gender**.

**Language**	**Stereotypicality of professions**	**Linguistic form**	**List**	**Participant gender**			
				**Female**		**Male**	
				***M***	***SD***	***M***	***SD***
German	Feminine professions	Masculine	1	3.97	0.74	3.97	0.84
			2	4.44	0.71	3.98	0.45
			3	4.03	0.84	3.60	0.53
		Word pairs	1	3.98	0.73	3.98	0.63
			2	4.06	1.06	3.88	1.02
			3	3.78	0.87	4.03	0.84
	Masculine professions	Masculine	1	4.58	0.69	4.30	0.66
			2	4.57	0.65	4.28	0.58
			3	4.07	0.84	3.67	0.69
		Word pairs	1	4.68	0.62	4.77	0.78
			2	4.43	1.03	4.53	1.28
			3	4.02	0.76	4.17	0.83
Italian	Feminine professions	Masculine	1	3.82	0.62	4.20	0.70
			2	4.10	0.83	4.14	0.76
			3	3.99	0.78	3.98	0.61
		Word pairs	1	4.05	0.54	3.83	0.46
			2	3.79	0.48	3.83	0.83
			3	4.03	0.88	3.83	1.29
	Masculine professions	Masculine	1	3.96	0.71	3.89	0.53
			2	4.25	0.72	4.30	0.62
			3	3.84	0.62	4.18	0.64
		Word pairs	1	4.12	0.61	4.30	0.43
			2	4.07	0.48	4.15	0.64
			3	4.07	0.62	3.85	0.91

*Ratings were given on a 7-point scale with higher scores indicating ascriptions of higher social status*.

### Estimated salary

**Figure 2:**
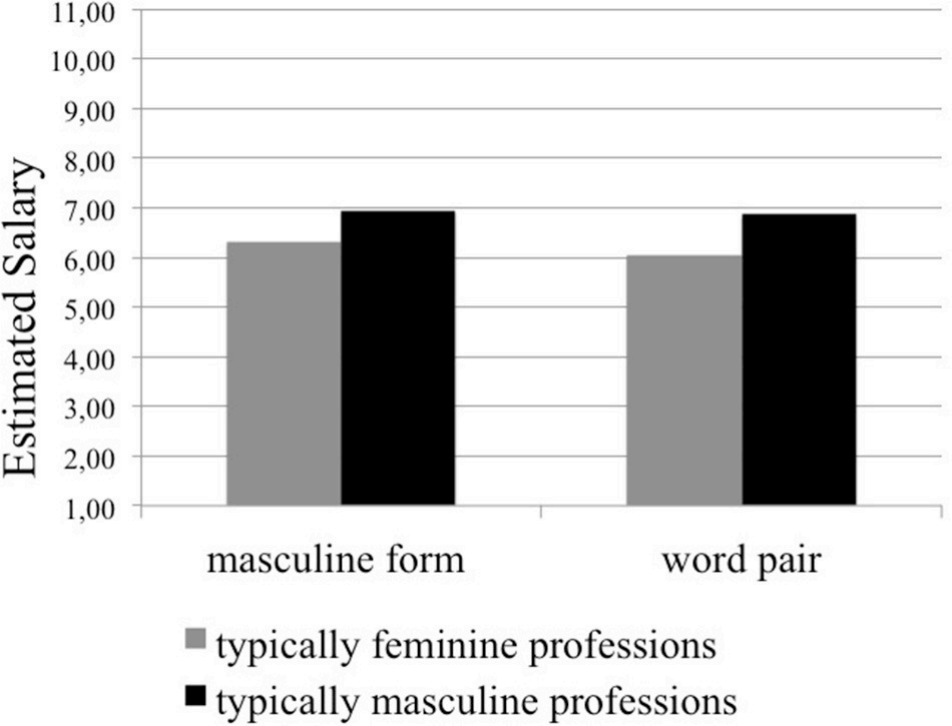
**Salary estimates for typically feminine and masculine professions**. Note that answers were given on a 11-point scale. Higher numbers indicate higher estimates of salary.

The main effect for linguistic form, *F*_(1, 359)_ = 5.85, *p* = 0.016, $\eta_{\text{p}}^{2}=0.02$, indicated that professions presented with masculine forms were believed to earn higher salaries than professions presented with word pairs (as predicted in Hypotheses 2). In addition, the interaction between stereotypicality of professions and linguistic form was significant, *F*_(1, 359)_ = 4.36, *p* = 0.037, $\eta_{\text{p}}^{2}=0.01$. Pairwise comparisons showed that feminine professions were estimated to have lower salaries than masculine professions in both linguistic conditions (masculine form: *p* < 0.001, $\eta_{\text{p}}^{2}=0.09$; word pairs: *p* < 0.001, $\eta_{\text{p}}^{2}=0.09$); however, salaries of feminine professions were estimated higher when designated with masculine forms than with word pairs (*p* = 0.003, η^2^ = 0.03). The salary ratings for masculine professions did not differ according to linguistic form (*p* = 0.416, η^2^ = 0.002). All means and standard deviations are reported in Table 4. These differences are displayed in Figure 2. For all other effects not involving linguistic form, please see Appendix C.

**Table 4:** **Means and standard deviations for estimated salary by stereotypicality of professions, linguistic form, and participant gender**.

**Language**	**Stereotypicality of professions**	**Linguistic form**	**List**	**Participant gender**			
				**Female**		**Male**	
				***M***	***SD***	***M***	***SD***
German	Feminine Professions	Masculine	1	6.20	0.87	6.10	1.20
			2	6.34	1.11	5.75	0.92
			3	6.47	1.63	6.50	0.41
		Word pairs	1	5.69	0.71	5.86	0.98
			2	5.87	1.09	5.81	1.31
			3	5.85	0.98	5.75	0.88
	Masculine Professions	Masculine	1	7.06	0.77	6.86	0.71
			2	7.34	0.68	7.25	0.82
			3	7.41	0.95	6.93	0.84
		Word pairs	1	7.05	0.87	6.73	1.01
			2	6.97	1.09	7.47	1.22
			3	6.85	0.72	6.88	1.24
Italian	Feminine Professions	Masculine	1	6.53	1.41	6.27	1.27
			2	6.31	0.95	6.29	1.42
			3	6.63	0.68	6.31	1.14
		Word pairs	1	5.97	1.54	6.50	0.94
			2	5.72	0.71	6.15	0.78
			3	6.37	0.87	5.78	1.37
	Masculine Professions	Masculine	1	6.41	1.11	6.36	1.60
			2	6.81	0.94	7.04	0.81
			3	6.96	0.82	6.88	0.99
		Word pairs	1	6.47	1.34	7.20	0.76
			2	6.61	0.80	6.70	0.63
			3	6.98	0.95	6.33	1.25

*Ratings were given on a 11-point scale with the midpoint (6) representing the national average salary. Higher scores indicate ascriptions of higher salary*.

### Women's visibility

A main effect of linguistic form, *F*_(1, 361)_ = 15.10, *p* < 0.001, $\eta_{\text{p}}^{2}=0.04$, indicated that women's visibility was higher with word pairs than with masculine forms. This is in line with Hypothesis 3. Furthermore, the interaction of linguistic form and list was significant, *F*_(2, 361)_ = 3.40, *p* = 0.034, $\eta_{\text{p}}^{2}=0.02$. Word pairs (vs. masculine forms) increased the visibility of women for List 2 (*p* = 0.028) and List 3 (*p* ≤ 0.001), but not for List 1. All means and standard deviations are reported in Table 5. For all other effects not involving linguistic form, please see Appendix C.

**Table 5:** **Means and standard deviations for women's visibility by stereotypicality of professions, linguistic form, list, and participant gender**.

**Language**	**Stereotypicality of professions**	**Linguistic form**	**List**	**Participant gender**			
				**Female**		**Male**	
				***M***	***SD***	***M***	***SD***
German	Feminine professions	Masculine	1	2.13	0.71	1.93	0.67
			2	2.38	0.79	1.98	1.18
			3	1.80	0.79	1.96	0.71
		Word pairs	1	2.53	0.65	2.23	0.68
			2	2.17	0.57	2.22	1.02
			3	2.40	0.66	2.05	0.87
	Masculine professions	Masculine	1	−1.84	0.77	−1.75	0.83
			2	−2.20	0.56	−2.18	0.64
			3	−2.39	0.47	−2.54	0.49
		Word pairs	1	−1.95	0.69	−1.75	0.96
			2	−2.16	0.73	−1.93	1.37
			3	−2.10	0.68	−2.17	0.72
Italian	Feminine professions	Masculine	1	1.99	0.88	2.27	0.79
			2	1.86	0.95	1.65	1.13
			3	1.45	0.77	1.16	0.82
		Word pairs	1	2.37	0.84	1.40	0.78
			2	2.06	0.89	2.13	0.94
			3	1.95	0.76	1.75	0.87
	Masculine professions	Masculine	1	−1.97	0.59	−2.09	0.53
			2	−2.44	0.58	−2.44	0.68
			3	−2.05	0.68	−2.09	0.69
		Word pairs	1	−1.99	0.77	−1.85	0.74
			2	−2.15	0.69	−2.18	1.29
			3	−1.87	0.38	−1.64	0.52

*The standardized scale for feminine and masculine professions was calculated by using z-scores of a 11-point and a 7-point scale. Higher values indicate higher visibility of women*.

### Ascribed competence

The ANOVA on competence revealed no significant effects involving linguistic form. All means and standard deviations are reported in Table 6. For all other effects not involving linguistic form, please see Appendix C.

**Table 6:** **Means and standard deviations for ascribed competence by stereotypicality of professions, linguistic form, list, and participant gender**.

**Language**	**Stereotypicality of professions**	**Linguistic form**	**List**	**Participant gender**			
				**Female**		**Male**	
				***M***	***SD***	***M***	***SD***
German	Feminine professions	Masculine	1	4.70	0.95	4.34	0.86
			2	5.37	0.91	4.87	0.73
			3	5.23	1.06	4.54	0.71
		Word pairs	1	5.03	0.96	4.55	0.55
			2	5.01	1.20	4.92	1.38
			3	5.07	1.04	5.08	0.71
	Masculine professions	Masculine	1	4.89	0.74	4.65	0.53
			2	5.21	0.84	4.83	0.70
			3	5.33	0.63	4.97	0.66
		Word pairs	1	5.34	0.70	5.36	0.85
			2	4.91	1.15	4.85	1.35
			3	4.92	0.95	5.29	0.90
Italian	Feminine professions	Masculine	1	4.57	0.99	4.89	1.11
			2	5.23	0.89	5.42	0.77
			3	4.74	0.87	4.71	0.68
		Word pairs	1	4.75	0.78	4.46	0.54
			2	4.58	0.92	5.03	1.16
			3	4.67	0.75	4.21	1.53
	Masculine professions	Masculine	1	5.02	0.78	5.61	0.85
			2	4.82	1.06	5.17	1.08
			3	4.55	0.75	4.99	0.56
		Word pairs	1	5.34	0.85	4.94	0.67
			2	4.79	0.86	5.10	0.82
			3	4.47	0.86	4.73	1.10

*Ratings were given on a 7-point scale with higher scores indicating ascriptions of higher competence*.

### Ascribed warmth

The ANOVA on ascribed warmth revealed a significant interaction between linguistic form and language, *F*_(1, 363)_ = 6.07, *p* = 0.014, $\eta_{\text{p}}^{2}=0.02$. This was qualified, however, by the three-way interaction of linguistic form, language and participant gender, *F*_(1, 363)_ = 6.64, *p* = 0.010, $\eta_{\text{p}}^{2}=0.02$. Pairwise comparisons within languages revealed the following for Italian: men perceived professions to be warmer when presented with masculine forms in comparison to women (*p* = 0.047, $\eta_{\text{p}}^{2}=0.01$) and in comparison to word pairs (*p* = 0.012, $\eta_{\text{p}}^{2}=0.017$). All means and standard deviations are reported in Table 7. For all other effects not involving linguistic form, please see Appendix C.

**Table 7:** **Means and standard deviations for ascribed warmth by stereotypicality of professions, linguistic form, list, and participant gender**.

**Language**	**Stereotypicality of professions**	**Linguistic form**	**List**	**Participant gender**			
				**Female**		**Male**	
				***M***	***SD***	***M***	***SD***
German	Feminine professions	Masculine	1	4.93	0.98	4.54	0.61
			2	4.53	0.58	4.47	0.31
			3	4.70	1.37	4.69	0.76
		Word pairs	1	5.23	0.90	5.04	0.60
			2	4.34	1.00	4.76	1.44
			3	4.57	1.26	4.87	0.63
	Masculine professions	Masculine	1	3.68	0.79	3.56	0.66
			2	4.12	0.56	3.70	0.67
			3	3.51	0.69	3.73	1.03
		Word pairs	1	3.87	0.88	4.11	0.63
			2	3.83	0.99	3.79	1.36
			3	3.86	1.05	4.16	1.03
Italian	Feminine professions	Masculine	1	4.27	1.14	5.01	1.07
			2	4.27	0.97	4.52	0.69
			3	4.13	0.69	4.38	0.51
		Word pairs	1	4.68	0.83	4.44	1.45
			2	4.08	0.49	4.11	0.54
			3	3.98	0.77	3.79	1.21
	Masculine professions	Masculine	1	3.50	0.99	4.11	1.28
			2	3.80	0.93	3.92	0.75
			3	3.55	0.83	3.44	0.89
		Word pairs	1	3.88	0.76	2.90	1.02
			2	3.64	0.64	3.76	0.67
			3	3.60	1.04	3.28	0.63

*Ratings were given on a 7-point scale with higher scores indicating ascriptions of higher warmth*.

## Discussion

The present research was designed to examine whether gender-fair language increases women's visibility and at the same time lowers status perceptions and salary estimates. We tested these effects in two languages with grammatical gender, Italian and German, within a single paradigm. Results mainly confirmed our hypotheses.

First of all, women's visibility increased for most professions when word pairs were used instead of masculine forms (see Hypothesis 3). This confirms the well-documented male bias in mental representation that is caused by masculine generics (e.g., Braun et al., 2005; Gabriel et al., 2008; Vervecken et al., 2013). With regard to the perceived social status of professions, typically feminine professions were ascribed significantly lower status than masculine professions, independent of linguistic form, which reflects the existing gender hierarchy (Eagly et al., 2000; Ridgeway and Correll, 2001). However, contrary to our expectations (see Hypothesis 1), professions did not lose in status when presented with word pairs compared to masculine forms. Instead, the difference in perceived social status between typically masculine and feminine professions increased when word pairs were used, as feminine professions slightly lost and masculine professions slightly gained in social status. This finding has to be treated with caution, however, because the differences between masculine forms and word pairs were not significant when typically feminine and typically masculine professions were treated separately. Salary estimates for feminine professions were also generally lower than for masculine professions. For typically feminine professions salary estimates were even lower when word pairs where used rather than masculine forms (see Hypothesis 2). In contrast, masculine professions were not affected by linguistic form. This pattern confirms Hypothesis 2 at least partially. Taken together, we can only partly confirm the detrimental effects of gender-fair word pairs on status-related measures (perceived social status and salary-estimates; e.g., Vervecken et al., 2013; Vervecken and Hannover, 2015).

Our exploration of ascribed competence and warmth showed that—in line with first results of Vervecken et al. (2015)—competence was not affected by linguistic form. This is an important finding in view of the fact that competence is highly relevant in professional contexts. For warmth, we observed an unexpected secondary effect, in that only male Italian participants were affected by linguistic form. They generally ascribed more warmth to professions designated in the masculine and less warmth to professions referred to with word pairs. In view of the means and of an effect on ascribed warmth reported by Vervecken et al. (2015), we would agree with the authors in the guess that word pairs shifted perceptions of warmth toward the midpoint of the scale and thus balanced warmth perceptions. We have no theoretical explanation for this result except for the fact that some studies found men to be more sensitive to linguistic forms than women in certain contexts (Braun et al., 2005). More research would have to be conducted to clarify this issue.

Results from correlational analyses revealed that status-related measures, though correlated with each other, are largely unrelated with ratings of women's visibility (i.e., gender-typicality). Thus, the two dimensions of status and gender-typicality appear to be independent of each other. This is in line with research by Glick et al. (1995) and Gottfredson (1996), which suggests that with respect to images of occupations status/prestige and gender-typicality are two orthogonal dimensions.

The present findings contribute to social role theory (Eagly, 1987; Eagly et al., 2000) in the following ways: Word pairs increase the inclusion of women in comparison to masculine forms and thus alter the perceived distribution of women and men across professional groups. In this way they affect occupational gender stereotyping. Our results are in accord with findings showing that the social status of professions does not readily translate into gender stereotypes ascribed to professions (Eagly and Steffen, 1984). Furthermore, given that fictitious and experimentally varied distributions of women and men in future professions can change ascribed gender stereotypes despite currently existing stereotypes (Koenig and Eagly, 2014), the use of word pairs might change occupational gender stereotyping on the long run, too. Further evidence for this idea comes from recent research which shows that linguistic forms in job advertisements for a typically masculine, high-status leadership position changed hiring decisions: Women and men were hired equally when word pairs (vs. masculine forms) were used in the respective job advertisement (Horvath and Sczesny, 2015). Moreover, girls' interest in typically masculine professions was found to be higher when these professions were presented with word pairs instead of masculine forms (Vervecken et al., 2015).

Our findings extend prior research by investigating, for the first time, whether beneficial and detrimental effects of gender-fair language on the social perception of professional groups emerge simultaneously: While previous studies focused mainly on individual professions (e.g., Braun et al., 2005; Gabriel et al., 2008) or individual targets (Merkel et al., 2012; Formanowicz et al., 2013), the current study sheds light on the social perception of a range of typically feminine and masculine professions. More importantly, our study was designed to capture both beneficial and detrimental effects of linguistic forms within a single paradigm. Earlier studies focused either on an increase in women's visibility (e.g., Braun et al., 2005; Gabriel et al., 2008) or on negative side-effects of gender-fair language (Mucchi-Faina, 2005; Merkel et al., 2012; Formanowicz et al., 2013). Our study shows that gender-fair language can simultaneously have positive effects (greater visibility of women) and negative effects (polarization of male-female differences in pay). Note, however, that the present study investigated only descriptive norms (how much status and pay does a given profession currently enjoy?) but not prescriptive norms (how much status and pay should a given profession enjoy?). Thus, it remains to be seen whether word pairs have detrimental effects on prescriptive norms as well.

One limitation of the present research is that we applied a between-participants design and presented participants either with masculine “generic” forms or with word pairs. Current language policies, however, demand the use of a whole range of gender-fair forms, including word pairs as well as other alternatives (for an overview of German gender-fair forms see Braun et al., 2005; Horvath, 2015). Consequently, speakers are likely to encounter many different forms in everyday life. Future research should therefore use a more ecologically valid approach and expose participants to diverse linguistic forms. In particular, future research should include gender-neutral expressions (e.g., German *Lehrkräfte*, teaching staff), which were not investigated here. In contrast to masculine forms and word pairs, gender-neutralizing forms make neither women nor men salient. It remains to be tested whether such neutral forms can increase women's visibility without reducing estimated salaries in comparison to word pairs. The finding that lower salaries are assumed for typically feminine professions designated with word pairs in contrast to masculine forms may simply reflect social reality, given that professions with a high percentage of women tend to be connected with lower salaries, lesser status, and lesser recognition in society. It is therefore conceivable that feminine-masculine word pairs designating typically feminine professions (vs. masculine forms) *automatically* activate knowledge about the gender wage gap or associations of men and wealth (Williams et al., 2010), while gender-neutral expressions do not. Such effects could be tested by measuring unconscious associations between professional groups (designated in the masculine, with word pairs or neutralizations) and the gender-wage gap, for example with implicit association tests.

Preliminary evidence has shown that in reference to typically feminine professions, feminine-only forms (e.g., *Kindergärtnerinnen*, kindergarden teachers, fem.) are more frequently used than masculine generics (*Kindergärtner*, kindergarden teachers, masc.) or word pairs (Chiarini, 2013; Hodel et al., 2013). Hence, future research should compare status and salary perceptions for typically feminine professions designated with word pairs compared to feminine forms. In this case, word pairs might actually cause an increase in estimated salaries and status perceptions, because masculine forms are here added to the feminine forms already in use. If this assumption should hold, it would again speak for a consistent use of gender-fair language, which in this case would mean replacing feminine-only forms with word pairs.

Now what are the practical implications of our results? Should word pairs be used to make language gender-fair and to support gender equality? The present findings indicate that, in German and Italian, language reform—and hence use of word pairs—is promising as they are likely to increase women's professional visibility on the one hand. On the other hand, word pairs in comparison to masculine forms may also lower estimated salaries of typically feminine professions. These effects appear to be inevitable for the time being. However, negative consequences of gender-fair language may diminish over time (Formanowicz et al., 2015; Gustafsson Senden et al., 2015). Furthermore, masculine generics are semantically ambiguous and thus problematic: they can refer to men only or to a group of women and men (Stahlberg et al., 2007). Therefore, we would recommend the use of gender-fair forms, such as word pairs or neutralizations in response to the question whether one should use gender-fair forms or masculine generics.

Taken together, our results on the social perception of professions indicate an increase of women's visibility with gender-fair language, but also a decrease in salary estimates of typically feminine professions. Although the latter effect is not negligible, social perceptions of status and competence do not suffer when word pairs are used.

### Conflict of interest statement

The authors declare that the research was conducted in the absence of any commercial or financial relationships that could be construed as a potential conflict of interest.

## Footnotes

^1^After measuring the dependent variables we also assessed participants' attitudes toward gender-fair language (Sczesny et al., 2015) and sexism (with the Ambivalent Sexism Inventory; Glick and Fiske, 1996). Since both attitude scales were correlated with the dependent as well as the independent variables, we could not use them as moderators, as had been intended, and thus do not report them here.
