# Supplementary material for: Does Gender-Fair Language Pay Off? The Social Perception of Professions from a Cross-Linguistic Perspective
Source: Front Psychol. 2016 Jan 21;6:2018. doi: 10.3389/fpsyg.2015.02018 (PMC4720790; doi:10.3389/fpsyg.2015.02018)
Supplement: Supplementary file 1 [file DataSheet1.pdf]

Appendix A

Pretest on the stereotypicality of professions

**English**	**German** (feminine-masculine word pairs)		**Italian** (feminine-masculine word pairs)	
		**Typically feminine professions**		
dancers	Tänzerinnen und Tänzer	*M* = 3.02, *SD* = .85	danzatrici e danzatori	*M* = 2.73, *SD* = .96
hairdressers	Friseurinnen und Friseure	*M* = 2.29, *SD* = 1.01	parrucchiere e parrucchieri	*M* = 2.97, *SD* = 1.08
interpreters	Dolmetscherinnen und Dolmetscher	*M* = 3.10, *SD* = 1.02	traduttrici e traduttori	*M* = 3.14, *SD* = 1.06
nutritionists	Ernährungsberaterinnen und Ernährungsberater	*M* = 2.32, *SD* = .88	nutrizioniste e nutrizionisti	*M* = 3.22, *SD* = .98
pharmacists	Apothekerinnen und Apotheker	*M* = 3.44, *SD* = 1.07	farmaciste e farmacisti	*M* = 3.38, *SD* = .95
psychologists	Psychologinnen und Psychologen	*M* = 3.12, *SD* = 1.05	psicologhe e psicologi	*M* = 3.08, *SD* = 1.16
tailors	Schneiderinnen und Schneider	*M* = 2.90, *SD* = 1.45	sarte e sarti	*M* = 2.86, *SD* = 1.09
		**Typically masculine professions**		
butchers	Fleischerinnen und Fleischer	*M* = 6.02, *SD* = .99	macellaie e macellai	*M* = 6.07, *SD* = .93
electricians	Elektrikerinnen und Elektriker	*M* = 6.22, *SD* = .76	elettriciste ed elettricisti	*M* = 6.51, *SD* = .94
brick layers	Maurerinnen und Maurer	*M* = 6.46, *SD* = .71	muratrici e muratori	*M* = 6.68, *SD* = .90
mechanics	Mechanikerinnen und Mechaniker	*M* = 6.12, *SD* = .93	meccaniche e meccanici	*M* = 6.51, *SD* = .95
computer scientists	Informatikerinnen und Informatiker	*M* = 5.81, *SD* = 1.08	informatiche ed informatici	*M* = 5.31, *SD* = 1.00
truckers	Lastwagenfahrerinnen und Lastwagenfahrer	*M* = 6.34, *SD* = .82	camioniste e camionisti	*M* = 6.27, *SD* = .87
engineers	Ingenieurinnen und Ingenieure	*M* = 5.39, *SD* = 1.11	ingegnere ed ingegneri	*M* = 5.08, *SD* = 1.18
		**Slightly masculine professions**		
bakers	Bäckerinnen und Bäcker	*M* = 4.98, *SD* = 1.13	panettiere e panettieri	*M* = 5.15, *SD* = 1.28
bankers	Bankerinnen und Banker	*M* = 5.07, *SD* = 1.03	banchiere e banchieri	*M* = 5.25, *SD* = 1.08
chefs	Köchinnen und Köche	*M* = 4.56, *SD* = .98	cuoche e cuochi	*M* = 4.62, *SD* = 1.12

farmers	Bäuerinnen und Bauern	*M* = 4.66, *SD* = 1.02	contadine e contadini	*M* = 4.90, *SD* = 1.09
mathematicians	Mathematikerinnen und Mathematiker	*M* = 5.37, *SD* = 1.02	matematiche e matematici	*M* = 4.78, *SD* = 1.18
physicians	Physikerinnen und Physiker	*M* = 5.39, *SD* = 1.12	fisiche e fisici	*M* = 4.93, *SD* = 1.11
**Gender-neutral professions**				
gynecologists	Gynäkologinnen und Gynäkologen	*M* = 4.39, *SD* = 1.32	ginecologhe e ginecologi	*M* = 3.56, *SD* = 1.05
historians	Historikerinnen und Historiker	*M* = 4.49, *SD* = 1.05	storiche e storici	*M* = 4.47, *SD* = 1.25
pediatricians	Kinderärztinnen und Kinderärzte	*M* = 3.85, *SD* = 1.04	pediatre e pediatri	*M* = 3.58, *SD* = .95
**Professions rated differently by the German and Italian pretest samples**				
letter carriers	Briefträgerinnen und Briefträger	*M* = 5.14, *SD* = .96	postine e postini	*M* = 4.14, *SD* = 1.12
librarians	Bibliothekarinnen und Bibliothekare	*M* = 3.49, *SD* = 1.16	bibliotecarie e bibliotecari	*M* = 3.54, *SD* = 1.16
salespersons	Verkäuferinnen und Verkäufer	*M* = 2.85, *SD* = .99	venditrici e venditori	*M* = 3.97, *SD* = .95
waiters	Kellnerinnen und Kellner	*M* = 3.34, *SD* = .96	cameriere e camerieri	*M* = 3.97, *SD* = .83

Appendix B

Target professions used in the main study, with stereotypicality of profession and distribution to list of professions.

	Stereotypicality of Profession	
List of professions	Typically feminine professions	Typically masculine professions
List 1	Hair dressers<br>Psychologists	Mechanics<br>Physisicts
List 2	Tailors<br>Interpreters	Electricians<br>Computer scientists
List 3	Dancers<br>Nutrition scientists	Truckers<br>Engineers

Appendix C
Further results, which do not involve linguistic form

MANOVA
The analysis revealed a main effect of stereotypicality of professions, $F(5, 354) = 699.84$, $p < .001$, $\eta^2_p = .91$, an interaction effect of stereotypicality of profession and participant gender, $F(5, 354) = 3.40$, $p = .005$, $\eta^2_p = .05$, stereotypicality of profession and list, $F(10, 710) = 11.79$, $p < .001$, $\eta^2_p = .14$, stereotypicality of profession and language, $F(5,354) = 3.52$, $p = .004$, $\eta^2_p = .05$, stereotypicality of profession, list and language, $F(10, 710) = 2.06$, $p = .026$, $\eta^2_p = .03$. Furthermore, a main effect of the list-factor $F(10, 710) = 3.64$, $p < .001$, $\eta^2_p = .05$, a main effect of language, $F(5, 354) = 5.56$, $p < .001$, $\eta^2_p = .07$, and an interaction of list and language, $F(10, 710) = 2.69$, $p = .003$, $\eta^2_p = .04$ reached significance.

ANOVAS
Perceived social status
The ANOVA for social status revealed a main effect of stereotypicality of profession, $F(1, 363) = 29.84$, $p < .001$, $\eta^2_p = .08$, indicating that feminine professions were ascribed lower social status ($M = 3.94$) than masculine professions ($M = 4.24$). The interaction effect of stereotypicality of profession and language was significant, $F(1, 363) = 7.72$, $p = .006$, $\eta^2_p = .02$. Pairwise comparisons indicated that feminine professions were ascribed lower social status than masculine professions in German ($M_{fem.prof.} = 3.99$ vs. $M_{masc.prof.} = 4.39$, $p < .001$, $\eta^2_p = .09$). Furthermore, the main effect of list, $F(2, 363) = 4.22$, $p = .015$, $\eta^2_p = .02$ reached significance, but was qualified by the interaction between stereotypicality of profession and list, $F(2, 363) = 3.62$, $p = .028$, $\eta^2_p = .02$. Pairwise comparisons indicated that feminine professions were ascribed significantly lower social status than masculine professions on list 1 ($M_{fem.prof.} = 3.97$ vs. $M_{masc.prof.} = 4.37$, $p \leq .001$, $\eta^2_p = .05$) and list 2 ($M_{fem.prof.} = 4.17$ vs. $M_{masc.prof.} = 4.33$, $p \leq .001$, $\eta^2_p = .04$), but not in list 3 ($M_{fem.prof.} = 3.95$ vs. $M_{masc.prof.} = 4.01$, $p = .332$, $\eta^2_p = .003$).

Estimated salary
The ANCOVA revealed a significant main effect for stereotypicality of professions, $F(1, 359) = 137.03$, $p \leq .001$, $\eta^2_p = .28$. Salaries of feminine professions ($M = 6.12$) were estimated to be lower than salaries of masculine professions ($M = 6.91$). Moreover, there was a significant interaction between stereotypicality of profession and language, $F(1, 359) = 17.90$, $p \leq .001$, $\eta^2_p = .05$. Feminine professions were estimated to have lower salaries both in German ($p \leq .001$, $\eta^2_p = .27$) and Italian ($p \leq .001$, $\eta^2_p = .07$); salary estimations for masculine profession were higher by German-speaking participants ($M = 7.08$) than by Italian-speaking participants ($M = 6.74$) ($p = .002$, $\eta^2_p = .03$).

Women's visibility
The ANOVA revealed a significant main effect for stereotypicality of professions, $F(1, 361) = 3489.12$, $p \leq .001$, $\eta^2_p = .91$. Women's visibility was higher in feminine professions ($M = 2.02$) than in masculine professions ($M = -2.07$). A significant main effect for language, $F(1, 361) = 7.81$, $p = .005$, $\eta^2_p = .02$, indicated that women's visibility was generally lower in Italian professions ($M = -.11$) than in German professions ($M = .03$). A main effect of the list factor, $F(1, 361) = 7.55$, $p \leq .001$, $\eta^2_p = .04$, indicated that women's visibility was generally higher in all professions on list 1 than in both list 2 ($p = .018$) and list 3 ($p \leq .001$). Lists 2 and 3 did not differ in this respect ($p = .809$).
The significant three-way-interaction between stereotypicality, language and list factor, $F(2, 361) = 3.81$, $p = .023$, $\eta^2_p = .02$, indicated that (a) women's visibility in feminine professions

was rated higher than in masculine professions in both languages across all lists (all *p*s ≤ .001). Only considering differences within languages, pairwise comparisons showed for German, that (b) women's visibility was rated higher for masculine professions on list 1 (e.g. mechanic & physicist) than for list 3 (e.g., truckers and engineers) (p = .002). In Italian, women's visibility of masculine professions on list 2 was rated higher than on list 3 (p = .012).

Ascribed competence

The ANOVA showed a significant main effect of stereotypicality of profession, $F(1, 363)$ = 14.26, $p \leq .001$, $\eta^2_p = .04$. Masculine professions (M = 4.99) were ascribed more competence than feminine professions (M = 4.87). All means and standard deviations are reported in Table 6. Moreover, the interaction effect between stereotypicality of profession and participant gender reached significance, $F(1, 363)$ = 6.65, p = .010, $\eta^2_p = .02$. This effect was driven by the fact that men ascribed typically feminine professions less competence (M = 4.82) than masculine professions (M = 4.97), $p \leq .001$. The significant interaction of stereotypicality and list, $F(2, 363)$ = 13.41, $p \leq .001$, $\eta^2_p = .07$, indicated that only for list 1 competence ascriptions were higher for feminine professions than for masculine professions ($p \leq .001$). The interaction also goes back to differences between competence ascriptions between lists: typically feminine professions from list 2 were ascribed more competence than feminine professions on list 1 (p = .008).

Ascribed warmth

The main effect for stereotypicality was significant, $F(1, 363)$ = 209.44, p = .001, $\eta^2_p = .37$. Typically feminine professions (M = 4.53) were perceived as warmer than masculine professions (M = 3.74). The main effect for language, $F(1, 363)$ = 13.71, $p \leq .001$, $\eta^2_p = .04$, indicated that German participants generally ascribed more warmth to professional groups (M = 4.27) than Italian participants (M = 3.96). Moreover, the interaction between stereotypicality and list was significant, $F(2, 363)$ = 6.96, $p \leq .001$, $\eta^2_p = .04$. All feminine professions were ascribed more warmth than masculine professions across all lists (all *p*s ≤ .001). Additionally, there were differences between the warmth perceptions of typically feminine professions across lists: feminine professions on list 1 were perceived to be warmer than professions on list 2 (p = .007) and list 3 (p = .010). There were no differences for masculine professions across lists.

---

[1] After measuring the dependent variables we also assessed participants' attitudes towards gender-fair language (Sczesny, Moser, and Wood, 2015) and sexism (with the Ambivalent Sexism Inventory; Glick and Fiske, 1996). Since both attitude scales were correlated with the dependent as well as the independent variables, we could not use them as moderators, as had been intended, and thus do not report them here.
